# A single-cell transposable element atlas of human cell identity

**DOI:** 10.1016/j.crmeth.2025.101086

**Published:** 2025-06-20

**Authors:** Helena Reyes-Gopar, Jez L. Marston, Bhavya Singh, Matthew Greenig, Jonah Lin, Mario A. Ostrowski, Kipchoge N. Randall, Santiago Sandoval-Motta, Nicholas Dopkins, Elsa Lawrence, Morgan M. O’Mara, Tongyi Fei, Rodrigo R.R. Duarte, Timothy R. Powell, Enrique Hernández-Lemus, Luis P. Iñiguez, Douglas F. Nixon, Matthew L. Bendall

**Affiliations:** 1 Division of Infectious Diseases, Department of Medicine, Weill Cornell Medicine, New York, NY 10021, USA; 2 Programa de Doctorado en Ciencias Biomédicas, Universidad Nacional Autónoma de México, Ciudad de México CDMX 04510, México; 3 Departamento de Genómica Computacional, Instituto Nacional de Medicina Genómica, Ciudad de México CDMX 14610, México; 4 Institute of Translational Research, Feinstein Institutes for Medical Research, Northwell Health, Manhasset, NY 11030, USA; 5 Department of Immunology and Immunotherapy, Icahn School of Medicine at Mount Sinai, New York, NY 10029, USA; 6 Yusuf Hamied Department of Chemistry, University of Cambridge, Cambridge CB2 1EW, UK; 7 Department of Immunology, University of Toronto, Toronto, ON M5S 3K3, Canada; 8 Department of Biological Sciences, San Jose State University, San Jose, CA 95112, USA; 9 Tri-Institutional MD-PhD Gateways Program, Weill Cornell Medicine, New York, NY 10021, USA; 10 Consejo Nacional de Ciencia y Tecnologia, IxM CONAHCYT, Ciudad de México CDMX 03940, México; 11 Centro de Ciencias de la Complejidad, Universidad Nacional Autónoma de México, Ciudad de México CDMX 04510, México; 12 Department of Molecular Medicine, Zucker School of Medicine at Hofstra/Northwell-Hofstra University, Hempstead, NY 11549, USA; 13 Department of Pharmacology, University of Cambridge, Cambridge CB2 1PD, UK; 14 Department of Social, Genetic & Developmental Psychiatry, Institute of Psychiatry, Psychology & Neuroscience, King’s College London, London SE5 8AF, UK

**Keywords:** single-cell RNA sequencing, transposable elements, transcriptomics, retrotranscriptomics, human endogenous retrovirus, long interspersed nuclear elements, machine learning, computational biology, Stellarscope, bioinformatics

## Abstract

Single-cell RNA sequencing (scRNA-seq) is revolutionizing the study of complex biological systems. However, most sequencing studies overlook the contribution of transposable element (TE) expression to the transcriptome. The quantification of locus-specific TE expression in scRNA-seq experiments is challenging due to their repetitive sequence content and poorly characterized annotations. Here, we developed a computational tool for single-cell transposable element locus-level analysis of scRNA sequencing (Stellarscope) that reassigns multimapped reads to specific genomic loci using an expectation maximization algorithm. Using Stellarscope, we built an atlas of TE expression in human PBMCs. We found that locus-specific TEs delineate cell types and define cell subsets not identified by standard mRNA expression profiles. Altogether, this study provides comprehensive insights into the influence of TEs in human biology at the single-cell level.

## Highlights

• Stellarscope quantifies locus-specific TE expression from scRNA-seq experiments
• Specific TE transcripts characterize different PBMC cell subtypes and human tissues
• Differential TE expression can group human immunocytes into cell subsets
• Plasmacytoid dendritic cells express high levels of specific HERV transcripts

## Motivation

Single-cell RNA sequencing (scRNA-seq) provides a high-resolution view of the transcriptome and promises a deeper understanding of cellular functions and heterogeneity. Transposable elements, which comprise a large proportion of mammalian genomes, are frequently ignored in transcriptome studies since they are not included in canonical gene models, and repetitive sequences complicate mapping and quantification. Furthermore, the sparsity of scRNA-seq data obfuscates statistical modeling for resolving mapping ambiguity. These challenges prompted us to develop an approach that accounts for both mapping uncertainty and low per-cell sequencing depth using a pooled Bayesian mixture model. Here, we present Stellarscope, an open-source package for quantifying the expression of specific transposable element loci in scRNA-seq data.

## Graphical abstract

**Figure:**
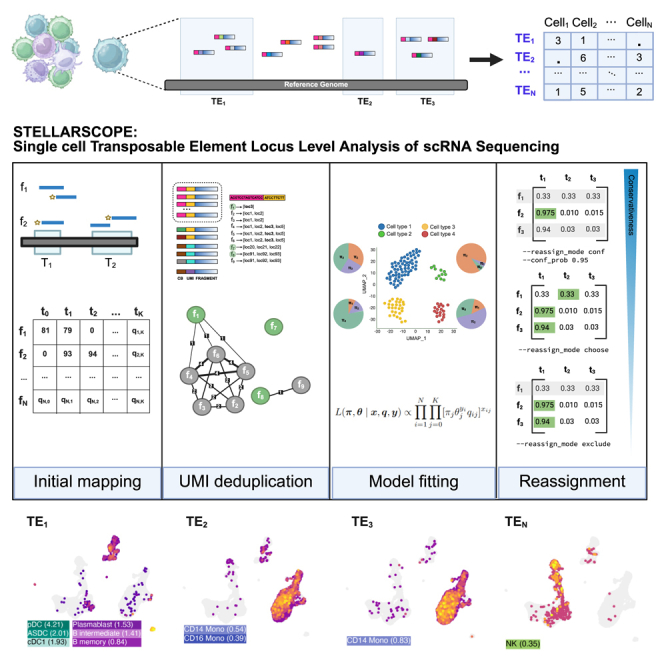

## Teaser

Reyes-Gopar et al. introduce Stellarscope, a computational approach for quantifying locus-specific transposable element expression in single-cell RNA sequencing data. The authors profile human PBMCs and reveal transposable elements marking specific cell subtypes. Stellarscope is available as an open-source package and promises to enable continued study of “genomic dark matter.”

## Introduction

The classification of human cells based on cell surface markers, and more recently, RNA expression, has led to a revolution in the understanding of cell function, lineage, and fate.^1^^,^^2^^,^^3^^,^^4^ High-quality markers correlate with characteristics and biological processes within the cell. However, these classifications have mostly been based on analyses of well-characterized reference gene models (canonical genes [CGs]), most of which are protein coding genes.^4^ A large fraction of the human genome is transposable elements (TEs), mobile genetic elements that are present in many nearly identical copies throughout the genome, and include human endogenous retroviruses (HERVs), remnants of ancient retroviral infections that invaded the germline and became fixed in human populations. They comprise almost 9% of the human genome^5^ and do not retrotranspose. Long interspersed nuclear element-1s (LINE1 or L1) also mobilize through an RNA intermediate and account for ∼17% of the genome,^5^ 80–100 of which are retrotransposition competent and contribute to genetic variation.^6^ In mammals, TEs impact embryonic development by providing *cis*-regulatory sequences and non-coding RNAs to contribute to cell stemness.^7^^,^^8^^,^^9^^,^^10^ Following embryonic development, the genomic accessibility of TEs is repressed as the genome accumulates epigenetic modifications to control their expression. In humans, thousands of TEs remain transcriptionally active in terminally differentiated cells,^11^^,^^12^ where they likely possess undetermined roles. Therefore, the accurate assessment of TE expression at a single-cell resolution is critical to their study in lineage development, cell subtype identification, and gene regulation.

Recent advances in computational biology have led to pipelines that can assess differential expression (DE) of TEs from bulk RNA sequencing (RNA-seq) data with locus specificity.^13^^,^^14^^,^^15^^,^^16^^,^^17^^,^^18^ However, there are several challenges in measuring TE expression in single cells.^18^ Complications regarding TE quantification in single-cell RNA-seq (scRNA-seq) may arise from rudimentary and underdeveloped TE gene models, low transcriptomic abundance, ambiguous mapping of repetitive sequences, and annotation variation.^18^^,^^19^^,^^20^ scRNA-seq approaches have fewer fragments sequenced per cell when compared to bulk RNA-seq approaches. As a result, TE-derived informative reads may not be retrieved for every sequenced cell, emphasizing the technological challenge of model-based TE quantification from single-cell datasets.

To address this challenge, we present a computational method for single-cell transposable element locus-level analysis of scRNA-sequencing, or Stellarscope. Stellarscope implements a statistical model that estimates locus-specific TE expression from droplet-based scRNA-seq data. By applying Stellarscope to a dataset of human peripheral blood mononuclear cells (PBMCs), we found that HERV and LINE1 transcripts can be reliably detected in scRNA-seq data, and that these transcripts contribute biologically relevant information to the transcriptome. We identified distinct PBMC subsets using locus-specific TE expression profiles compared to CGs alone.

Using Stellarscope, we accurately calculated single-cell TE expression profiles in differentiated hematological cell types and used these profiles to improve the identification of distinct cell subtypes when compared to CGs alone. By providing a single-cell-resolution multi-scale analysis of TE expression in PBMCs, we further illustrate the emerging association between TE activity and human cell identity. With these findings, we argue that the continued study of TE-derived cell-type markers will likely improve upon cell-type characterization techniques and find functional roles for sequences derived from the “dark matter” of the human genome.

## Results

### Stellarscope design

**Figure 1:**
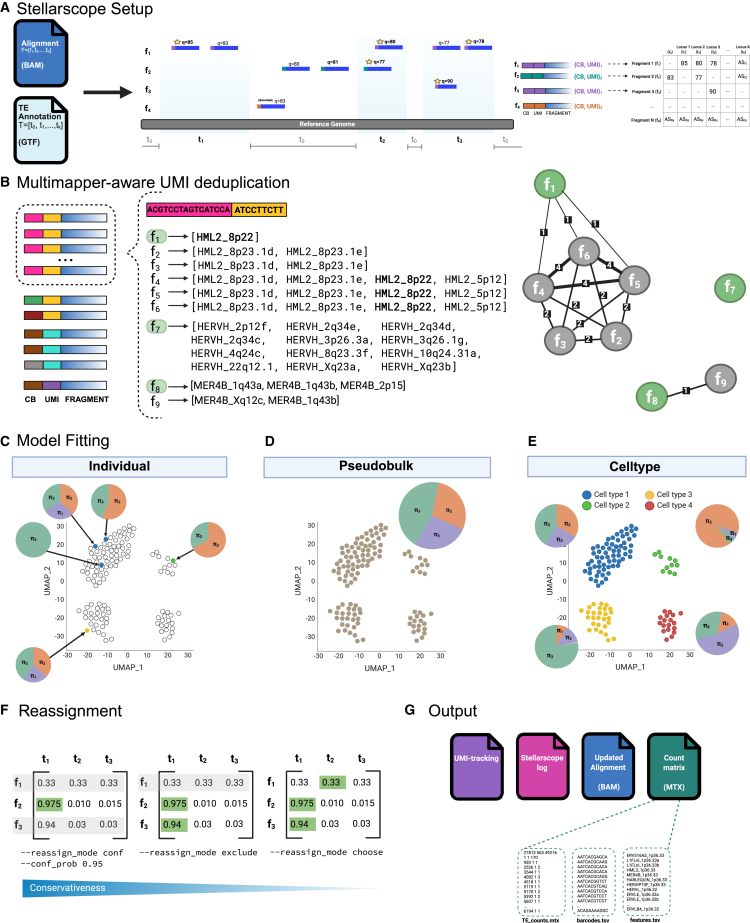
Stellarscope—Single-cell transposable element locus-level analysis of scRNA sequencing (A) Stellarscope setup. Alignments from a binary alignment map (BAM) file are intersected with a TE annotation to obtain an initial weight matrix. Alignment metadata, including the cell barcode (CB) and unique molecular identifier (UMI), are used for filtering and stored internally. (B) Multimapper-aware UMI deduplication. Fragments with the same CB + UMI combination are identified. For each CB + UMI group, an undirected weighted graph is constructed with fragments as nodes and shared alignments as edge weights. A connected component in this graph represents a set of fragments with shared alignment positions. For each component in the graph, the most informative read is selected as the representative, while others are discarded. (C–E) Model fitting. A Bayesian mixture model is fitted to the deduplicated weight matrix using an expectation maximization algorithm. Alignment weights are pooled, and parameters are estimated for (C) each cell individually, (D) across all cells, or (E) according to cell-type label. (F) Reassignment. The posterior assignment probabilities are used to determine the most probable read assignment using the selected reassignment strategy. (G) Stellarscope reports UMI counts for each cell and locus in the MTX format. Additional outputs include updated alignments (BAM file) and reporting information for UMI deduplication and model fitting.

Stellarscope is a computational tool that quantifies the expression of specific TE insertions in scRNA-seq experiments (Figure 1). The primary challenge with locus-specific TE quantification is that sequencing reads originating from TE-derived transcripts align ambiguously to multiple genomic locations. Our approach uses a Bayesian mixture model that is fitted using an expectation maximization (EM) algorithm, a solution that has been widely used for repetitive sequence quantification in bulk RNA-seq.^13^^,^^15^^,^^18^^,^^21^ In contrast to bulk RNA-seq, scRNA-seq data are more sparse and more complex, thus motivating the development of additional approaches for single-cell TE analysis. TE quantification based on single-cell long read RNA-seq (i.e., CELLO-seq^22^) avoids some problems with poorly annotated TE gene models and mapping uncertainty, but depends on data availability and lacks the throughput of short-read sequencing. In developing Stellarscope, we chose to focus on droplet-based short-read scRNA-seq since this method has been used to profile over 100 million cells,^23^ and we expect this technology to be highly relevant for developing TE expression atlases.

Existing approaches have been proposed for the quantification of TE expression in single cells. scTE^24^ quantifies TEs at the subfamily level by allocating reads to TE metagenes. SoloTE^25^ uses a similar approach for locus-specific quantification, summarizing at the subfamily level when accurate locus-specific assignment is not possible. SCIFER^26^ quantifies L1 elements at single-locus resolution using uniquely mapping reads only, requiring validated annotations from matched bulk RNA-seq. We refer to these approaches as filtering approaches, as they do not aim to resolve the correct location of multimapping reads but instead discard or summarize ambiguous reads. In contrast, statistical approaches have been found to be more accurate than filtering approaches.^27^ Ad hoc implementations of statistical models have been reported,^28^^,^^29^ yet model-based approaches have not been widely implemented as bioinformatic tools.

Stellarscope performs locus-specific TE quantification using four main stages and introduces two key innovations specific to scRNA-seq data: multimapper-aware PCR deduplication and pooling modes for model fitting (Figures 1B–1E). Standard practice for the removal of PCR duplicates considers reads to be duplicates when they share the same unique molecular identifier (UMI) and genomic mapping location, which is inherently problematic when the mapping location is ambiguous. Stellarscope implements an approach that considers the full set of possible mapping locations for each read (Figure 1B). Duplicates are inferred when reads share some of the same mapping locations, and only the most informative duplicated read is retained (Figure 1B).

Our initial strategy for profiling locus-specific TEs in single cells was to simply split the dataset into individual cells and independently fit separate models for each barcoded cell. This approach minimizes assumptions as the final assignments depend solely on informative reads from the same cell (Figure 1C). In practice, there are not enough informative reads within each cell due to small per-cell sequencing depth and characteristically low TE abundance. To address this challenge, we implement a model that pools information across cells to resolve read assignments within cells. The “pseudobulk” pooling model estimates one set of model parameters for all cells (Figure 1D), while read membership probabilities and final assignments are determined at a single-cell level. The implicit assumption is that TE expression patterns in the sample are representative of expression in each cell and are suitable when cellular heterogeneity is low. To address situations with high heterogeneity, we implemented the “celltype” pooling model, which fits a separate model for each cell type label (Figure 1E). This pooling strategy assumes that TE expression patterns are similar among cells with the same label and are not dependent on sample-level TE expression. The labels must be provided as input and can be determined using supervised or unsupervised approaches.

Stellarscope reports TE expression as a UMI count matrix; a reassigned binary alignment map (BAM) file containing alignment posterior probabilities may also be generated (Figure 1G). Stellarscope is designed to be user-friendly, adaptable to different analysis objectives, and provides output compatible with downstream single cell analysis tools.

### The retrotranscriptome of human PBMCs at single-cell resolution

**Figure 2:**
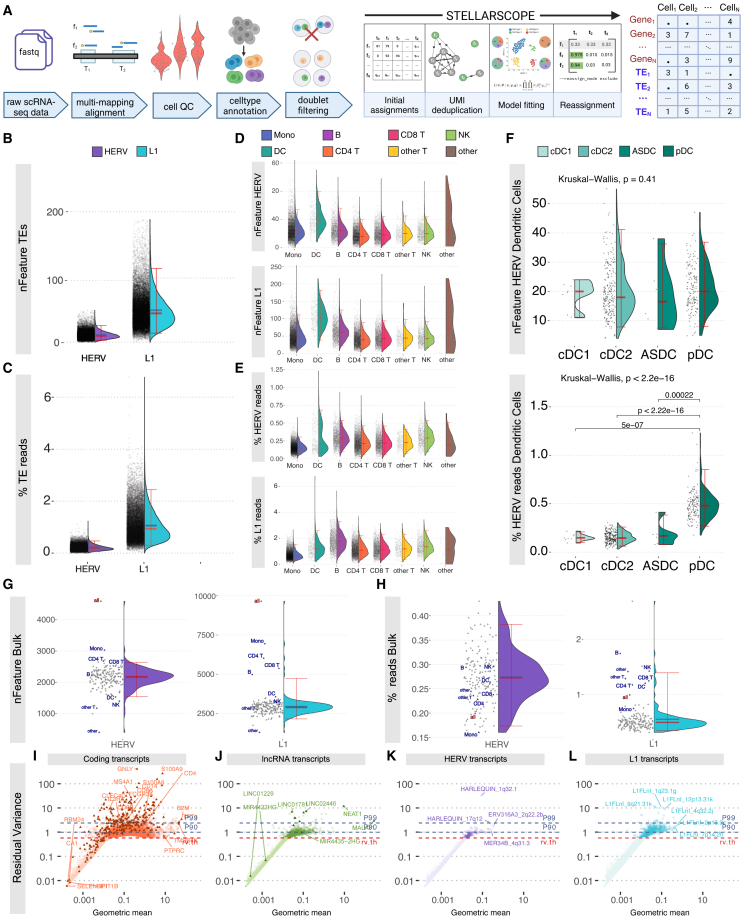
Stellarscope determines the retrotranscriptome of human leukocytes at single-cell resolution (A) Analysis pipeline for obtaining PBMC scRNA-seq matrix counts: alignment to reference genome retaining multimappers; cell quality control using adaptive thresholds to identify and exclude outlier cells; cell-type annotation using Azimuth^31^ and human PBMCs^32^; multiplet removal with Scrublet^33^; final CB input to Stellarscope with the alignment for ambiguous read reassignment and TE feature counting. (B–F) The distribution of the number of HERV or L1 features detected (B, D, and F) and the percentage of UMI counts assigned to HERV or L1 features (C, E, and F). Distributions are shown across all cells (B and C), partitioned by cell type (D and E) and for dendritic cell subtypes (F). (G and H) (G) Number of detected TE features and (H) percentage of TE reads from 157 PBMC samples using bulk RNA-seq (gray) and pseudobulk aggregation of scRNA-seq data by cell type (blue) and by total cells (red). (I–L) Residual variance (RV) versus geometric mean for features subset by transcript biotype: coding (I), lncRNA (J), HERV (K), and L1 (L). The 90^th^ and 99^th^ percentile RV values (P90, P99) are shown as blue dotted lines, and the RV threshold used to determine highly variable features is shown in red. Triangles indicate marker genes used in cell-type annotation.

We assessed TE contribution to single-cell transcriptomes by profiling TE-derived transcripts in human PBMCs. Sequencing reads were aligned to the human genome (hg38) reporting up to 500 high-scoring alignments for multimapping reads (STARsolo^30^). Multimapping reads were reassigned to their most probable location using Stellarscope. UMI counts for TEs were corrected for overlap with CG exons and joined with CG counts for downstream analysis (Figure 2A).

We detected TE expression and identified a median of 12 HERV features and 57 L1 features per cell (Figure 2B), accounting for an average of 0.21% and 1.05% of UMI counts per cell, respectively (Figure 2C). We used reference transcriptome mapping^34^ to investigate distinct TE expression patterns in PBMC subtypes. Dendritic cells (DCs) expressed more TE features than other cell types (Figure 2D), but their percentage of TE UMI counts (TE load) was not similarly elevated (Figure 2E). HERV load in dendritic cells was bimodally distributed, indicating distinct levels of HERV expression within the same cell type (Figure 2E). We did not find significant differences in the number of HERV features among conventional DCs (cDC1, cDC2), AXL+ DCs, and plasmacytoid DCs (pDCs) (*p* = 0.41), but pDCs had significantly higher HERV loads compared to other DCs (Kruskal-Wallis, *p* < 2.2e−16) (Figures 2F, S1, and S2 for all cell subtypes). Overall, TE expression was quantifiable in single-cell expression profiling data, and the detected TE expression signal may be applied to further distinguish cell types.

We compared TE expression measurements obtained using bulk and single-cell approaches from the same tissue type. Bulk RNA-seq data were obtained from 157 PBMC healthy donors aged 20–74 years.^35^ Sequencing reads were aligned using equivalent parameters; TE expression was quantified using Telescope^13^ with identical TE annotations. Pseudobulk expression profiles were aggregated from single-cell UMI counts for the entire sample and each cell type. We detected a higher number of both HERV and L1 features in single-cell data (Figure 2G) consistent with prior findings.^29^ This is possibly explained by differences in sequencing depth: increased sequencing depth increases the chances of detecting low abundance transcripts. The pseudobulk dataset contained >142 million UMI counts, while bulk samples had fewer than 15 million fragments on average. HERV load in bulk samples (range: 0.16%–0.43%, mean = 0.28%) was comparable to pseudobulk HERV load (0.21%) (Figure 2H), while L1 load in bulk (range: 0.32%–2.69%, mean = 0.52%) was less than pseudobulk L1 load (1.00%) for nearly all samples (Figure 2H). This disparity could be explained by differences in the genomic locations between the two TE classes: L1 elements are more frequently found overlapping CG introns or exons than HERVs and are thus more likely to be detected as part of CG pre-mRNA transcripts.

Next, we asked whether TE expression is the result of technical noise or whether TE features exhibit high biological heterogeneity and are informative for ascribing biological characteristics to individual cells.^36^ We used the residual variance (RV), the remaining variance after correcting for technical effects,^36^ to identify relevant TEs and to compare to CGs with known biological relevance. The RV of canonical protein-coding genes ranges between 1 and 10 (Figure 2I), while long non-coding RNAs (lncRNAs) tended to have less variance (Figure 2J). TEs have lower RV (between 1 and 2) compared to CGs (Figures 2K and 2L). The RV of L1 elements was greater than the RV of HERVs, but both TE classes were in the same range as lncRNAs (Figure 2J). We compared TEs with established marker genes, a subset of CGs considered useful for distinguishing cell types and subtypes. Some established markers had unexpectedly low RV, below the RV of some TEs. These observations demonstrate considerable overlap among the RV distributions of coding genes, lncRNAs, and TEs, suggesting that TEs share some of the same statistical properties as CGs and are a frequently overlooked source of biological heterogeneity.

### Distinct PBMC subtypes are identified using locus-specific TE expression profiles compared to CGs alone

**Figure 3:**
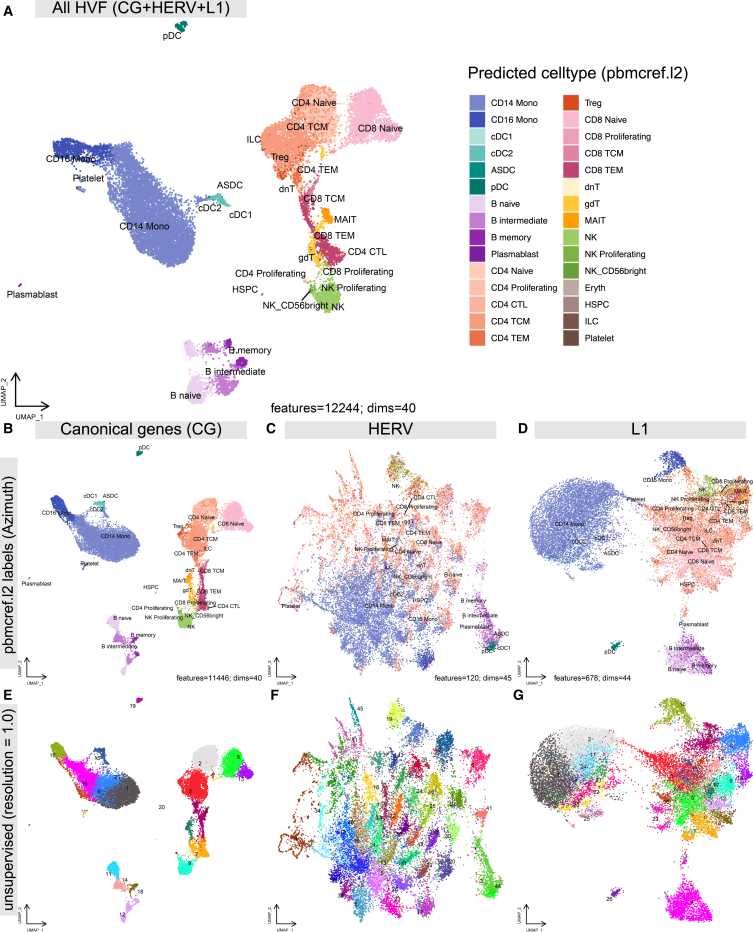
TE features inform cell relationships and subtypes (A) UMAP constructed using all highly variable features (HVFs), including CG, HERV, and L1. Cells are colored according to predicted cell type (human PBMC reference, celltype.l2). UMAP based on top 40 principal components for 11,750 HVFs. (B–G) UMAP representations constructed using HVF subsets: CG only (B and E), HERV (C and F), and L1 (D and G). Cells are colored according to predicted cell type (B–D) or based on unsupervised cluster label using resolution = 1 (E–G). The number of features and principal components used are in the lower right of each plot.

Resolution of gene expression at the single-cell level can uncover previously undescribed cell subsets and better characterize the physiology of lowly abundant cell types.^37^^,^^38^ Because prior studies used established gene models excluding TEs, we investigated whether HERV/L1 expression offered cell classifications beyond those based on CGs. Using different sets of highly variable features (HVFs) including or excluding TEs, we performed dimensionality reduction (DR) using principal-component analysis and uniform manifold approximation and projection (UMAP). The complete set of HVFs (all-HVFs, including CG, HERV, and L1; 12,244 features) yields a projection that distinguishes major PBMC lineages and cell types (Figure 3A). The UMAP found using only CGs (CG-HVFs; 11,446 features) (Figure 3B) was visually similar to the all-HVF projection since CG-HVFs make up most of the all-HVF set (93%) and account for most of the biological variability.

UMAPs based only on HERV-HVFs (120 features) were not visually similar to the CG-based projections and describe different similarity patterns among cells (Figure 3C)**.** The HERV-only UMAP shows distinctions between major PBMC cell types (monocytes, B cells, DCs, natural killer [NK] cells), but cell subtypes were not distinguishable. For example, T cells are found in a particular region of the UMAP, but there was no clear separation between CD4^+^ and CD8^+^ T cells. HERV-HVFs account for fewer features and less variance than CG-HVFs, resulting in less information overall. Collectively, this indicates that the expression of HERVs alone is not informative enough to distinguish between established PBMC cell subtypes. Despite this, there is structure in the HERV expression patterns driving similarities among cells, as evidenced by groupings of cells in UMAP space that may enhance CG-based cell-type characterizations. These groupings may be driven by a small number of HERV-HVFs and may have biological relevance reflecting cell states or processes distinct from established cell-type identities. In contrast to the HERV-based UMAP, the L1-HVF projection (678 features) was visually similar to the all-HVF UMAP, albeit with less definition within cell types (Figure 3D).

To identify TE-based cell subtype clusters, we performed unsupervised community detection with the Leiden algorithm^39^ (resolution = 1.0) on HERV and L1-HVF subsets, testing the hypothesis that retrotranscriptome data could reveal previously undefined scRNA-seq cell subcategories. With HERV-HVFs, many of the clusters identified did not correspond to cell subtype labels (Figures 3E–3G), although some labels formed multiple clusters and may reflect cell subtypes defined by HERVs. This result provides evidence that TE transcript expression can be unique to certain cell-type transcriptomes and therefore contribute to cell identity. These findings are a compelling argument for the inclusion of the non-canonical TE transcriptome in analyzing scRNA-seq data.

### HERV subfamily expression

**Figure 4:**
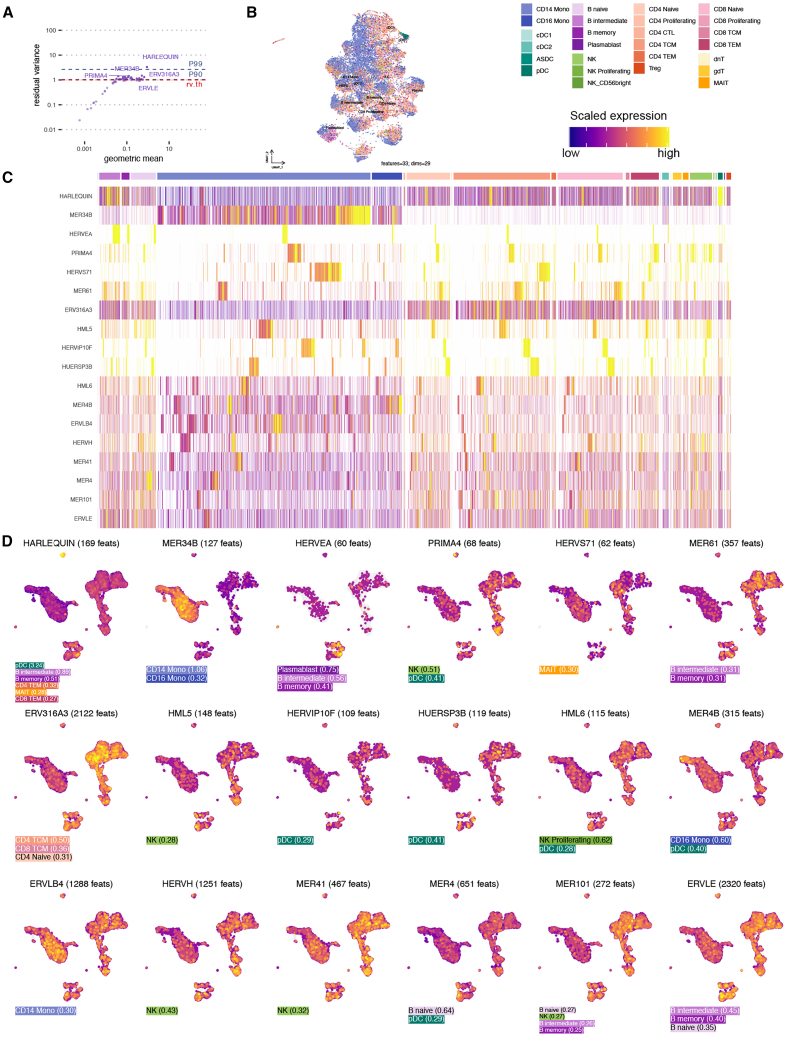
Expression of HERV subfamilies in PBMC (A) RV of aggregated HERV subfamily features versus geometric mean. The 90^th^ and 90^th^ percentile RV values (P90, P99) are shown as blue dotted lines, and the RV threshold used to determine highly variable features is shown in red. (B) UMAP using only aggregated HERV subfamily features (35 features, 31 principal components). Cells colored by Azimuth-predicted PBMC cell-type identities (predicted.celltype.l2). (C) Heatmap showing relative expression in each cell for 20 HERV subfamily features with significant differential expression. Rows represent HERV subfamily features (names on *y* axis). Columns represent individual cells with predicted cell subtypes shown above. Features are ordered by hierarchical clustering. Cells are grouped by subset and then hierarchically clustered within each subset. (D) Feature plots showing relative expression for 20 HERV subfamily features with significant differential expression. Cells are colored by scaled HERV expression (see legend), and cells with no detection are colored gray. Cell positions are identical to those in Figure 3A (all-HVF UMAP). The lower left of each plot is annotated with the cell subsets in which the feature is significantly upregulated and the average LFC in parentheses.

TE subfamilies are groups of related TE loci represented by one model or consensus sequence. We examined HERV subfamily expression by aggregating Stellarscope locus-specific UMI counts. We identified 33 highly variable subfamilies with RV > 1; the HARLEQUIN and MER34B subfamilies had the greatest RV (RV = 3.34 and 1.49, respectively) (Figure 4A). Locus-specific elements from these subfamilies were also highly variable, suggesting that subfamily aggregation retains some locus-specific TE expression heterogeneity; however, some variance is lost, especially for loci with extremely high RV.

We performed DR using HERV subfamily features. As expected, the UMAP visualization based on CGs together with HERV subfamilies recapitulated the UMAP obtained using CGs with HERV loci, since CGs represent the majority of the features and variability. Projections based only on HERV subfamily features (Figure 4B) appeared similar to the locus-specific HERV UMAP (Figure 3C). Several subtypes that formed distinct groupings using locus-level features were not distinct in the subfamily UMAP, including CD14 monocytes, CD16 monocytes, and NK cells.

We performed DE testing for each predicted cell subtype (celltype.l2) compared to all other cells. We identified 18 subfamilies with significant differences in one or more cell subtypes (adjusted *p* value [padj] < 0.05, average log_2_ fold change [LFC] > 0.25) (Figures 4C and 4D). Some families, such as MER4B, ERVLB4, and HERVH, were expressed across all cell subtypes, with modest effect sizes. MER34B is significantly upregulated in CD14 (LFC = 1.06) and CD16 monocytes (LFC = 0.32), with lower expression in other cell types. pDCs had the most subfamily-level activity, with seven subfamilies upregulated: HARLEQUIN, PRIMA4, HERVIP10F, HUERSP3B, HML6, MER4B, and MER4. We identified three B cell-specific markers (HERVEA, MER61, and ERVLE) and one T cell marker (ERV316A3).

### PBMC subtypes are characterized by specific HERV loci expression

**Figure 5:**
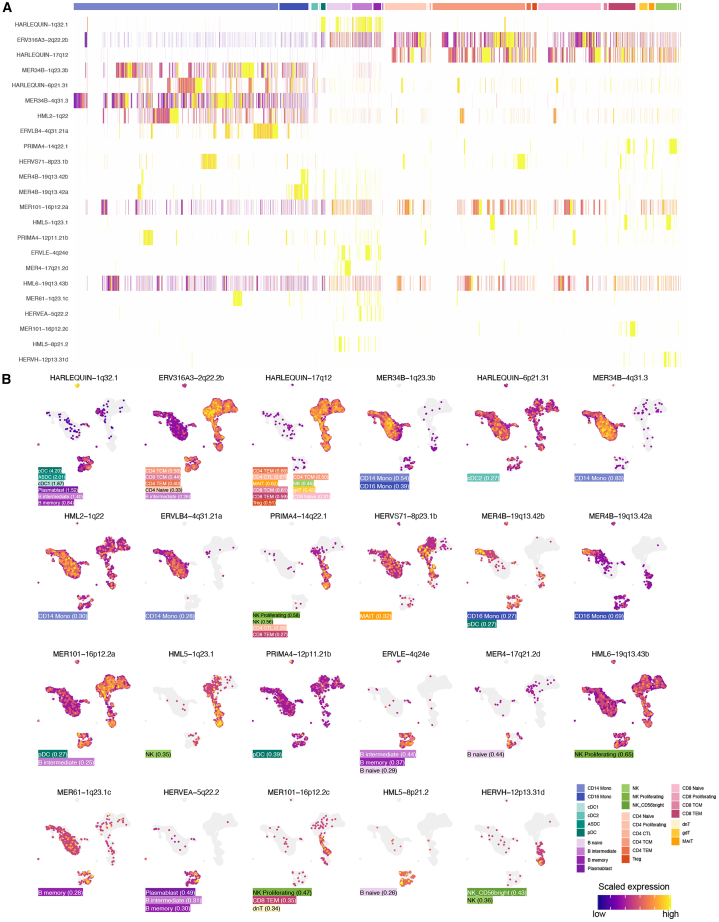
Expression of locus specific HERV features characterizes immune cell subtypes (A) Heatmap showing relative expression in each cell for 27 HERV features with significant differential expression. Rows represent HERV features (names on *y* axis). Columns represent individual cells with predicted cell subsets shown above (colored by cell type). Features are ordered by hierarchical clustering. Cells are grouped by subset, then hierarchically clustered within each subset. (B) Feature plots showing relative expression for 27 HERV features with significant differential expression in ≥1 cell subset comparisons. Each plot is titled with the feature name; cells are colored by scaled HERV expression (see legend); no detection is colored gray. Cell positions are identical to those in Figure 3A (all-HVF UMAP). The lower left of each plot is annotated with the cell subsets in which the feature is significantly upregulated, and the average LFC is in parentheses.

Cell classification typically uses markers like surface proteins and RNA expression.^2^^,^^40^ While most markers are protein-coding genes, some lncRNAs show high sensitivity in transcriptomic studies.^41^ The utility of TE-derived RNAs as markers has not previously been demonstrated, partially due to technological challenges with assaying the expression of specific TE insertions. We find that locus-specific HERV transcripts are distinctly expressed in differentiated hematological cell types (Figure 5A). Transcriptional differences among cells correlated with known cell types, including subtypes within T cells, B cells, and monocyte lineages. Overall, we identified 59 significant tests representing 28 distinct HERV loci with differences in expression in one or more cell subtypes when compared with all other cells (padj < 0.05, average LFC >0.25) (Figure 5B).

In pDCs, the relatively high HERV load (Figure 2F) involved many differentially expressed loci, including four significant HERV markers: HARLEQUIN-1q32.1, MER4B-19q13.42b, MER101-16p12.2a, and PRIMA4-12p11.21b. One locus, PRIMA4-12p11.21b (LFC = 0.39) was only significant in pDCs, but it appears to be lowly expressed in many cell types (Figure 5B). MER101-16p12.2a was significantly upregulated in pDCs and B intermediate cells, while MER4B-19q13.42b was shared with CD16 monocytes.

Lymphocyte subtypes were marked by significant upregulation of 13 HERV elements. Generally, markers were shared by multiple subtypes but were exclusive to a single cell type. ERVLE-4q24e, HERVEA-5q22.2, and HARLEQUIN-1q32.1 (see above) each marked multiple B cell subtypes, while MER4-17q21.2d and HML5-8p21.2 were specific to naive B cells and MER61-1q23.1 to memory B cells. ERV316A3-2q22.2b and HARLEQUIN-17q12 appear to be pan T cell markers, although the former is also significantly expressed in intermediate B cells and lowly expressed across all cell types. Three markers, HML5-1q23.1, HML6-19q13.43b, and HERVH-12p13.31d, are specific to NK cells, while two elements are significantly expressed in both NK and T cell subtypes (PRIMA4-14q22.1 and MER101-16p12.2c). The lack of subtype-specific markers in lymphocytes may indicate that HERV expression plays a more important role in cell-type identity as opposed to subtype-specific programs.

We found HARLEQUIN-1q32.1 to be a significant marker for six subtypes, including three DC subtypes and three B cell subtypes. Notably, this feature had the greatest RV of all TE features and ranked in the 99^th^ percentile for all features (Figure 2K). Comparing pDCs with all other cells, we found that HARLEQUIN-1q32.1 (LFC = 4.20) had the largest effect size of any TE or CG feature including established pDC markers E2-2/TCF4^42^ (LFC = 3.77) and IRF8^43^ (LFC = 3.45). HARLEQUIN-1q32.1 overlaps an isoform of RHEX (regulator of hemoglobinization and erythroid cell expansion) and pseudogene CH17-84K15.2. RHEX was not expressed in these data, while CH17-84K15.2 is significantly expressed in pDC (padj = 2.19e−171, LFC = 0.88) and plasmablasts (padj = 8.66e−3, LFC = 0.32). Improved characterization of HARLEQUIN-1q32.1 transcription is needed to further elucidate the role of this locus in pDCs.

### Stellarscope quantifies locus-specific TE expression across diverse droplet-based protocols

**Figure 6:**
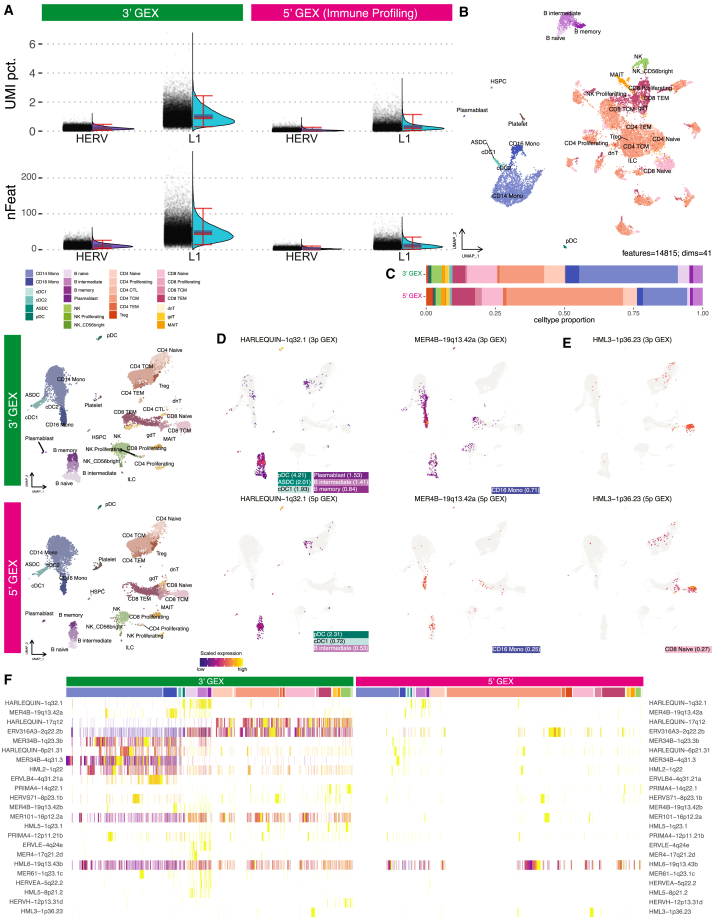
Comparison of 5′GEX with 3′GEX in PBMC (A) Violin plots comparing percentage of UMI counts and number of features from TE loci in PBMCs between 3′GEX and 5′GEX protocols. (B) UMAP of all HVFs (CG, HERV, L1). Cells are colored by Azimuth-predicted cell types from the Human BioMolecular Atlas Program PBMC reference. The UMAP is based on the top 41 principal components for 14,815 HVFs. (C) Cell-type proportion comparison between 3′GEX and 5′GEX datasets. Bar width indicates proportion of cells per cell type. (D and E) Feature plots showing relative expression for three HERV features with significant differential expression in the 5′GEX dataset compared with the same features in the 3′GEX. Both datasets are projected into the human PBMC UMAP reference space (left). Feature plots (right) show scaled HERV expression per cell with significantly upregulated cell subsets annotated (LFC in parentheses). (F) Heatmap showing relative expression for 23 HERV features with significant differential expression in either dataset. Rows represent HERV features; columns represent cells grouped by subset and hierarchically clustered within each subset.

Stellarscope can be broadly applied to different droplet-based sequencing technologies for locus-specific TE quantification. We validated our findings using an immune profiling dataset of ∼20,000 PBMCs from a healthy donor (STAR Methods). Unlike the 3′ gene expression (3′GEX) assay described above, the immune profiling protocol (5′GEX) sequences the 5′ end of polyadenylated transcripts. We found lower per-cell sequencing depth in 5′GEX compared with 3′GEX, with post-filtering medians of 4,376 and 5,533 UMIs per cell, respectively (Figure S3). We expect this reduced sequencing depth to have a disproportionate effect on lowly expressed genes, including TEs, and this resulted in important differences in downstream analysis. HERVs and L1s both account for a smaller percentage of the total UMI counts per cell in 5′GEX, with a mean load of 0.07% and 0.33%, respectively (Figure 6A). DR of HVFs revealed less cohesion among cell type groupings when visualized using UMAP (Figure 6B). Cell type proportions also differed among the two protocols (Figure 6C).

5′GEX yielded only three locus-specific HERV markers with significant DE in one or more cell subtypes when compared with all the other cells (padj <0.05, average LFC >0.25). HARLEQUIN-1q32.1 was significantly upregulated in pDC, cDC1, and B intermediates, and MER4B-19q13.42a was upregulated in CD16 monocytes (Figure 6D). These two loci were also significant in 3′GEX, although significance levels and effect sizes were smaller in 5′GEX. The third significant locus in 5′GEX, HML3-1p36.23, is significantly upregulated in CD8 naive T cells, but it was not significant in 3′GEX (Figure 6E). We compared 5′GEX expression of the 21 HERV markers that were identified only with 3′GEX (Figures 6F and S4). Some markers (e.g., ERV316A3-2q22.2b, MER34B-1q23.3b) had similar cell-type expression patterns in both assays, but they failed to reach significance in the 5′ dataset. Other loci, such as ERVLE-4q24e, MER4-17q21.2d, and MER101-16p12.2c, had no expression in the 5′ assay. Although many loci demonstrated similar trends, we did not find significant agreement between the datasets, suggesting that 5′GEX is underpowered.

### TEs are expressed across human tissue types

We characterized TE expression across diverse healthy human tissues using single-nucleus data from the Genotype-Tissue Expression (GTEx) project.^44^ Our analyses included breast mammary tissue, prostate, heart left ventricle, gastrocnemius skeletal muscle, esophagus muscularis, and lung.

**Figure 7:**
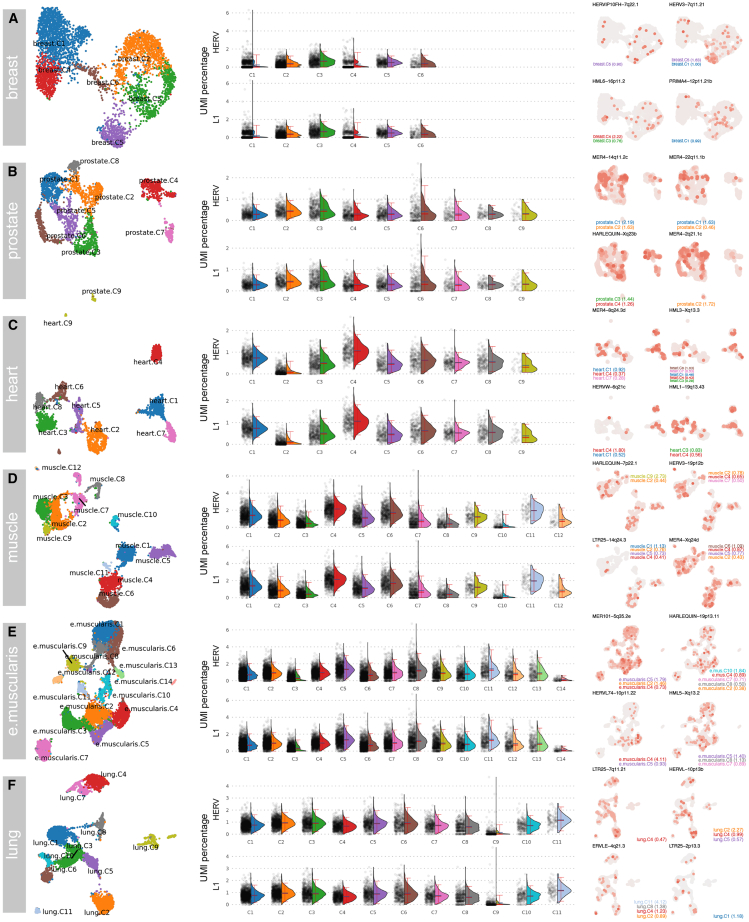
Locus-specific TE expression across human tissues HERV and L1 expression analysis across human tissues: (A) breast, (B) prostate, (C) heart, (D) skeletal muscle (gastrocnemius), (E) esophagus muscularis, and (F) lung. For each tissue: (left) UMAP with unsupervised cell clustering; (center) combined violin and dot plots showing percentage of UMIs from TEs (HERV and L1) across clusters; (right) UMAP plots of top four markers (by padj), with expression levels indicated by color intensity. The cluster for which each TE feature serves as a marker is indicated below each UMAP.

After quality control, alignment, and Stellarscope TE quantification with correction for overlapping CG annotations, we found expressed TEs in all the tissues exhibiting tissue-specific patterns (Figure 7). The proportion of cellular transcriptomes derived from TEs ranged from 0% to 6% of a cell’s UMIs, demonstrating heterogeneity within and between tissues.

In breast, TE expression profiles were highly variable. C1 cells had high levels of both L1 and HERV expression (up to 6% of total UMIs), while C1 and C4 displayed bimodal patterns, with some cells showing virtually no TE expression. HERV3-7q11.21 and PRIMA4-12p11.21b emerged as specific markers for C1, while HML6-16p11.2 characterized C3 and C4.

Prostate tissue showed more uniform TE expression, with the exception of C6, where a subset of cells displayed high L1 and HERV expression. Despite comprising few cells, C9 maintained detectable expression of both L1 and HERV loci. C2 was marked by three members of the MER4 family (MER4-14q11.2c, MER4-22q11.1b, and MER4-2q21.1c), while HARLEQUIN-Xq23b marked both C3 and C4.

Heart tissue analysis showed C4 to be distinct, both visually (in the UMAP) and by having the highest TE levels. Conversely, C2 consistently showed low TE expression. TE expression varied substantially across skeletal muscle clusters; C10 showed the lowest TE load, while C4 displayed the highest. TE expression was more uniform in esophagus muscularis, except for C3 and C14 (very low/absent expression) and C8 (highest TE UMI proportion, up to 6%).

Lung tissue presented a unique expression profile. C9 was heterogeneous, containing both the highest and lowest TE-expressing cells, with most cells lacking detectable expression. These findings highlight the ubiquitous yet tissue-specific nature of TE expression, with distinct HERV loci expression observed at the cluster level that may reflect tissue-specific regulatory mechanisms or functional roles of TEs in defining tissue cellular identity.

### Comparison of Stellarscope with existing approaches

We compared Stellarscope with two TE quantification software packages: scTE^24^ and soloTE.^25^ Each program was used to quantify HERV expression using the same 3′ PBMC alignment and equivalent HERV annotation files adapted for each program. Since neither scTE nor soloTE attempt to “rescue” ambiguously mapped fragments, we expected these methods to report lower UMI counts than Stellarscope; however, Stellarscope reported the fewest UMIs of the three methods. Stellarscope reported a total of 283,004 UMIs across all cells, while scTE reported 387,856 UMIs, and soloTE reported 1,589,071 UMIs (676,316 at the locus level). To determine whether these estimates are feasible, we calculated the expected bounds on the true UMI count based on unique and ambiguously mapping reads in the original alignment file (STAR Methods). Only Stellarscope was within these bounds (254,964–355,895), while scTE and soloTE estimates exceeded the upper bound. Next, we compared cell-level UMI count estimates to expected bounds calculated for each cell. scTE estimates skewed high; UMI counts were within bounds in only 30.2% of cells, and 49.7% exceeded the upper bound. soloTE estimates exceeded the upper bound in 97.4% of cells, while the remainder were within bounds. Stellarscope was within expected bounds in 89.1% of cells, while only 0.1% of cells exceeded the upper bound. Stellarscope UMI counts were lower than expected in 10.8% of cells due to Stellarscope’s default requirement of 20% alignment overlap with the TE annotation. This default setting is designed to be conservative but can be adjusted through a user-defined parameter. We recalculated the expected bounds using 20% overlap criteria and found that UMI counts are underestimated in only 1.5% of cells.

## Discussion

Cell identity classification is iterative and adapts to new technologies and characterization of lowly abundant cell types.^2^^,^^40^ However, existing techniques for cell-type identification ignore potential contributions from TEs. We present an scRNA-seq-based computational tool and pipeline for characterizing cell identity based on the expression of L1 and HERV elements. We demonstrate that TEs can be identified from scRNA-seq data at a locus-specific level, and their expression signatures can be incorporated into existing cell-type classification to potentially identify previously undescribed cell subtypes distinct from those defined by CG markers.

Stellarscope filters alignments according to passing barcodes, identifies and removes PCR duplicates using a multimapper-aware UMI deduplication approach, and fits a Bayesian mixture model to the deduplicated weight matrix using an EM algorithm. Importantly, due to the relatively small sequencing depth of TEs per cell, pooling models enable the utilization of information across cells for resolving ambiguous reads.

Using human PBMC scRNA-seq data, we found that TEs contributed an average of 2.7% of the total features detected in each cell. TEs tended to have lower RV (between 1 and 2) compared to CGs (between 1 and 10), but we found TE features with greater RV than established marker genes, suggesting that despite their lower abundances, TE RNAs possess similar cell-type-specific properties as CG RNAs. Under this assumption, HERV and L1 expression is less likely to be the result of random noise or aberrant transcription but instead points toward a deliberate expression of TE transcripts.

Using different sets of HVFs that include or exclude TEs, we performed DR and unsupervised clustering. The complete set of HVFs (including CG, HERV, and L1) yielded a representation that clearly distinguishes major PBMC lineages and cell types. Unsupervised clustering using only HERV-HVFs identified expression similarities as subclusters within broader cell types, including NK cells and CD4^+^ T cells, while B cells and pDCs formed distinct clusters of cells using HERV features alone.

DCs expressed more TE features than other cell types, with a median of 19 HERV and 102 L1 features detected per cell. Interestingly, pDCs had significantly higher HERV loads than other DC subtypes, matched by many differentially expressed loci. One locus, PRIMA4-12p11.21b, was unique to pDCs. Our single-cell profiling provides insights into cell identities by uncovering unique TE transcripts delineating known cell subtypes.

TE quantification can be performed using either subfamily-level or locus-specific approaches. Stellarscope uses a locus-specific approach to pinpoint the precise genomic location of TE expression and maximize the amount of informative data. However, given the low overall TE abundance and per-cell sequencing depth in single-cell libraries, aggregating by subfamily may help avoid problems with dropout or normalization. Subfamily-level analysis may be more suitable for studying TE regulatory networks or epigenetic regulation, while locus-specific analysis is needed to examine the local genomic context of individual TE insertions or determine RNA or peptide products from a particular locus. We recommend considering these approaches based on the scientific questions posed.

Comparison with scTE and soloTE revealed that both methods tend to overestimate TE expression, while Stellarscope’s UMI count estimates tend to fall (89.1%) within the expected bounds. Estimates from Stellarscope are less than the expected lower bound in about 10% of cells due to the default overlap requirement used in Stellarscope. However, if we use the same criteria to calculate bounds, the percentage of estimates within bounds goes up to 98.0%. For scTE and soloTE, the extreme differences between the expected bounds and estimated UMI counts is concerning, as users of these programs will greatly overestimate the amount of TE expression. A detailed comparison using simulated and empirical data is needed to establish their accuracy and statistical performance.

As the known role of TEs in biology grows, with major contributions noted in human development, aging, neurodegenerative diseases, and cancer, understanding how single cells express TEs is critical to understand their roles in biology and human diseases. Our study pioneers a method for integrated analysis of comprehensive single-cell genomics and tissue datasets and provides knowledge and opportunities to unravel the complexities of cell identities.

### Limitations of the study

Locus-specific studies of TE expression have long been encumbered by poorly characterized TE gene models and TE-derived transcripts.^18^^,^^19^^,^^20^ Fundamentally, RNA-seq-based techniques measure gene expression by counting the number of RNA molecules that originate from annotated genomic regions and thus depend on high-quality functional annotations for meaningful inference. Stellarscope solves the problem that arises when there are several possible regions where a fragment may have originated but cannot determine whether the TE-derived fragment is part of a larger transcript or whether the molecules observed are “actively” or “passively” expressed. Different types of assays, such as long-read sequencing or C1 CAGE, are needed to determine full-length gene models for TEs. Providing these types of improved annotations as input for Stellarscope will enable further inference beyond basic expression quantification and lead to improved biological insights.

In this study, the TE annotation was developed based on genomic annotations of TE sequences (STAR Methods) and not transcript-based gene models. TE transcription is believed to be more complex,^29^^,^^45^^,^^46^ but reference TE gene models are not available. However, our TE quantification approach is methodologically sound in terms of quantifying the annotation provided, and we designed Stellarscope to accommodate any gene model provided in gene transfer format.

When combining Stellarscope UMI count matrices with other expression data, it is important to consider that Stellarscope quantifies TE loci independently from other quantification methods. Thus, observations may be counted multiple times if the fragment overlaps features in both annotations, and the combined count matrix will overestimate the actual number of UMIs observed. For this study, we implemented a correction procedure downstream of Stellarscope that removes TE counts that are assigned to CGs, thus avoiding artifactual observations in our analysis.

Finally, the insights gained from the human datasets in this study will need to be validated with additional individuals and molecular approaches. Further work is needed to develop experimental tools to mark the expression of TE open reading frames (including specific antibodies) and locus-specific TE probes.

## Resource availability

### Lead contact

All further information, requests for access to resources, or clarifications should be directed to Dr. Matthew L. Bendall (mlb4001@med.cornell.edu).

### Materials availability

This study did not generate new or unique reagents.

### Data and code availability

• This study utilized only publicly available data. 3′ and 5′ scRNA-seq datasets for 20,000 human PBMCs are available from 10x Genomics. Single-nucleus data from healthy human tissues are available from GTEx version 9 (dbGaP: phs000424). Bulk RNA-seq data from 157 human PBMCs are available from NCBI (GEO: GSE193141).
• Stellarscope is freely available under an open-source license on GitHub: https://github.com/nixonlab/stellarscope. The version used in this study (version 1.4) has been released as a Zenodo archive: https://doi.org/10.5281/zenodo.15377350. TE annotation retro.hg38.v1 is available on GitHub: https://github.com/mlbendall/telescope_annotation_db and as a Zenodo archive: https://doi.org/10.5281/zenodo.6423053.
• Any additional information required to re-analyze the data reported in this study is available from the lead contact upon request.

## Acknowledgments

We thank Cedric Feschotte, Ethel Cesarman, Ulrike Lange, and members of their labs for discussions about TEs. We thank Nicholas Liotta for testing and feedback on the Stellarscope software. The work was supported by US National Institutes of Health (NIH) grants CA260691, CA206488, R21HG011513, R56AG078970, and UM1AI164559. M.L.B. is supported in part by the Department of Medicine Fund for the Future program at Weill Cornell Medicine sponsored by the Elsa Miller Foundation. J.L.M. was supported in part by a Medical Scientist Training Program grant to the Weill Cornell-Rockefeller-Sloan Kettering Tri-Institutional MD-PhD Program (T32GM007739). T.R.P. is supported by an MRC (UKRI) New Investigator Research Grant (MR/W028018/1) and by the 10.13039/501100000272National Institute for Health Research (NIHR) Maudsley Biomedical Research Centre at South London and Maudsley National Health Service (NHS) Foundation Trust and King’s College London. R.R.R.D. and T.R.P. are supported by a 10.13039/100016210Psychiatry Research Trust grant. The content and views expressed are solely the responsibility of the authors and do not necessarily represent the official views of the funding bodies, including NIH, NHS, NIHR, or the Department of Health and Social Care. The funders had no role in the design of the study; in the collection, analyses, or interpretation of data; in the writing of the manuscript; or in the decision to publish the results.

## Author contributions

Conceptualization, H.R.-G., J.L.M., D.F.N., and M.L.B.; methodology, H.R.-G., J.L.M., B.S., M.G., J.L., K.N.R., S.S.-M., T.F., E.H.-L., L.P.I., and M.L.B.; software, H.R.-G., J.L.M., B.S., M.G., S.S.-M., L.P.I., and M.L.B.; formal analysis, H.R.-G., J.L.M., B.S., M.G., S.S.-M., L.P.I., and M.L.B.; writing – original draft, H.R.-G., J.L.M., B.S., M.G., J.L., M.A.O., K.N.R., S.S.-M., N.D., E.L., M.M.O., T.F., L.P.I., D.F.N., and M.L.B.; writing – review & editing, H.R.-G., J.L.M., M.G., J.L., S.S.-M., N.D., E.L., T.R.P., R.R.R.D., L.P.I., D.F.N., and M.L.B.; supervision, M.A.O., E.H.-L., L.P.I., D.F.N., and M.L.B.; funding acquisition, J.L.M., M.A.O., S.S.-M., T.R.P., E.H.-L., D.F.N., and M.L.B.

## Declaration of interests

The authors declare no competing interests.

## STAR★Methods

### Key resources table

**Table:** 

REAGENT or RESOURCE	SOURCE	IDENTIFIER
**Deposited data**		

scRNA-seq, 20k human PBMCs, 3′GEX	10x Genomics	https://www.10xgenomics.com/datasets/20-k-human-pbm-cs-3-ht-v-3-1-chromium-x-3-1-high-6-1-0
scRNA-seq, 20k human PBMCs, 5′GEX	10x Genomics	https://www.10xgenomics.com/resources/datasets/20-k-human-pbm-cs-5-ht-v-2-0-2-high-6-1-0
GTEx v9 snRNA-seq	GTEx	dbGaP: phs000424
PBMC bulk RNA-seq	Morandini et al.^35^	GEO: GSE193141

**Software and algorithms**		

Stellarscope (1.4)	This paper	https://doi.org/10.5281/zenodo.15377350
STARsolo (2.7.10b)	Dobin et al.^30^	https://github.com/alexdobin/STAR
Seurat (4.4.0)	Hao and Hao et al.^32^	https://satijalab.org/seurat
scTransform (0.4.1)	Hafemeister et al.^36^	https://github.com/satijalab/sctransform
MAST	Finak et al.^47^	https://bioconductor.org/packages/MAST
Telescope (1.0.3)	Bendall et al.^13^	https://github.com/mlbendall/telescope
scater (1.28.0)	McCarthy et al.^48^	https://bioconductor.org/scater
scrublet (0.2.3)	Wolock et al.^33^	https://github.com/swolock/scrublet
Azimuth (0.4.6)	Butler et al.44	https://github.com/satijalab/azimuth
scTE (1.0)	He et al.^24^	https://github.com/JiekaiLab/scTE
soloTE (1.09)	Rodríguez-Quiroz et al.^25^	https://github.com/bvaldebenitom/SoloTE

**Other**		

TE Annotation (retro.hg38.v1)	Bendall et al.^13^	https://doi.org/10.5281/zenodo.6423053
Human PBMC reference	Human BioMolecular Atlas Program, Hao and Hao et al.^32^	https://azimuth.hubmapconsortium.org/references

### Method details

#### Stellarscope: Single-cell Transposable Element Locus Level Analysis of scRNA sequencing

##### Multimapper-aware UMI deduplication

For Stellarscope, we developed a multimapper-aware UMI deduplication approach for identifying and removing PCR duplicates (Figure 1B). First, reads are binned according to their error-corrected barcode and UMI sequence (hereafter referred to as “UMI”) reported in the BAM file. Next, for each UMI, we determine which reads originate from the same genomic location. Since multimapped reads have multiple possible locations, we determine whether there is intersection between their sets of mapping locations. We also consider that, due to incomplete mapping, two reads may not have any mapping locations in common but may both intersect with a third read (i.e., Figure 1B, reads f1, f2, and f4). To facilitate identification of reads with intersecting sets of mapping locations, we construct an undirected graph with nodes corresponding to reads and edges connecting reads when both reads have an alignment to the same locus (Figure 1B). Within this graph, a component (or connected subgraph) represents a set of reads with intersecting mapping locations; we assume that these are PCR duplicates originating from the same molecule. A graph containing multiple components occurs when the same UMI is used to label distinct molecules due to low UMI pool complexity. For each component, the most informative duplicated read is selected as a representative. The result of this stage is a corrected weight matrix with UMI duplicates removed.

##### Single cell reassignment mixture model

Stellarscope implements a generative model of single cell RNA-seq that rescales alignment probabilities for independently aligned reads based on the cumulative weights of all alignments to each feature. Fundamentally, the probability that a given alignment is the “true” alignment increases when the total supporting information for that feature is greater. The model and notation follow from Bendall et al. 2019.^13^ Each sequencing fragment is comprised of three parts that are tracked by our model: 1) $F=\left[f_{1},f_{2},\ldots ,f_{N}\right]$, the set of N observed cDNA sequences from the originating feature; 2) the corresponding cell barcodes $B=\left[b_{1},b_{2},\ldots ,b_{N}\right]$, where $b_{i\;}=b_{j\;}$ for all i and j that originate from the same cell; and 3) a Unique Molecular Identifier (UMI) $U=\left[u_{1},u_{2},\ldots ,u_{N}\right]$ for each template molecule. Let $C=\left[c_{1},c_{2},\ldots ,c_{M}\right]$, be the set of M cells that are included in the model. Cells are categorized *a priori* into subsets, or “pools”, depending on the chosen pooling mode. Let $Ρ=\left[P_{1},P_{2},\ldots ,P_{D}\right]$ be the set of D pools, and let $P=\left[p_{1},p_{2},\ldots ,p_{M}\right]$, be an indicator mapping each cell to the pool to which it belongs, $\forall_{i}\;p_{i}\in Ρ$. For individual pooling mode, each cell is in a separate pool ($\forall_{i}\;p_{i}=c_{i}$). For pseudobulk pooling mode, all cells are in the same pool ($\forall_{i}\;p_{i}=1$). For celltype pooling mode, the pool assignment for each cell is provided as input for the model. For each pool, we estimate the abundance parameter $\pi_{P}=\left[\pi_{P_{0}},\pi_{P_{1}},\ldots ,\pi_{P_{K}}\right]$ representing the proportion of total fragments originating from each of K annotated features. In addition, we estimate the reassignment parameter $\theta_{P}=\left[\theta_{P_{0}},\theta_{P_{1}},\ldots ,\theta_{P_{K}}\right]$ representing the proportion of ambiguous fragments generated by each feature. Thus, the probability of observing fragment $f_{i}$ with cell barcode $b_{i}$ is given by:

$$\mathrm{Pr}\left(f_{i},b_{i}|\pi_{P},\theta_{P},q_{i}\right)=\sum_{j=0}^{K}\pi_{P_{j}}{\theta_{P_{j}}}^{y_{i}}q_{ij}$$

where P is the pool containing cell barcode $b_{i}$ ($p_{b_{i}}$), $\pi_{P}$ and $\theta_{P}$ are pool-specific parameters, $q_{i}$ is a vector of mapping qualities for $f_{i}$, and $y_{i}$ is an indicator where $y_{i}=1$ if $f_{i}$ is ambiguously aligned and $y_{i}=0$ otherwise.

As in earlier work, we formulate a mixture model accounting for uncertainty in the initial fragment assignments. Let ${x_{P}}_{i}=\left[{x_{P}}_{i0},{x_{P}}_{i1},\ldots ,{x_{P}}_{iK}\right]$ be a set of partial assignment (or membership) weights for fragment $f_{i}$ in pool P. If $f_{i}$ did not originate from pool P ($p_{b_{i}}\neq P$), then ${{\forall_{j\;}x}_{P}}_{ij}=0$; otherwise $\sum_{j=0}^{K}{x_{P}}_{ij}=1$ and ${x_{P}}_{ij}=0$ if $f_{i}$ does not align to $t_{j}$. We assume that ${x_{P}}_{i}$ is distributed according to a multinomial distribution with success probability $\pi_{P}$. Intuitively, $x_{ij}$ represents our confidence that $f_{i}$ was generated by feature $t_{j}$. The complete data likelihood across all pools is

$$L\left(\pi ,\theta |x,q,y,P\right)\propto \prod_{P}^{Ρ}\prod_{i=1}^{N}\prod_{j=0}^{K}{\left[{\pi_{P}}_{j}{\theta_{P}}_{j}^{y_{i}}q_{ij}\right]}^{{x_{P}}_{ij}}$$

##### Model selection

Stellarscope reports several model selection criteria for choosing the pooling mode or celltype assignment that best fits a particular dataset. The complete data log likelihood (lnL) of the data given the fitted model parameters is given above. The Bayesian information criterion (BIC) and Akaike information criterion (AIC) are criteria that penalize models that estimate more parameters. The BIC and AIC are

BIC=kln(n)−2lnLandAIC=2k−2lnL

where k is the number of estimated parameters and n is the number of observations. For each pool, two parameters ($\pi_{P}$ and $\theta_{P}$) are estimated for each observed transcript j; k is given by summing the number of paramters in each pool. The number of observations is equal to the number of aligned reads after UMI deduplication.

##### TE annotations

We used an annotation of 28,513 HERV and L1 elements. HERV elements were assembled for 60 HERV subfamilies from RepeatMasker annotations as described by Bendall et al.^13^ L1 element annotations for full length and fully length nearly intact elements were obtained from L1base^49^ and reformatted in Gene Transfer Format (GTF). The annotation scripts, and supporting documentation are deposited and available at https://github.com/mlbendall/telescope_annotation_db/tree/master/builds/retro.hg38.v1.

#### scRNA-seq and snRNA-seq sequence data

##### PBMC 3′ gene expression (3′GEX)

Sequencing reads for ∼20K PBMCs were downloaded from 10X Genomics. PBMCs were obtained from a healthy female donor aged 25–30 and sequenced by 10x Genomics using the Chromium Next GEM Single Cell 3′ HT Reagent Kit v3.1.

##### PBMC 5′ immune profiling (5′GEX)

Sequencing reads for ∼20K PBMCs were downloaded from 10X Genomics. PBMCs were obtained from a healthy male donor aged 30–35 and sequenced using the Chromium Next GEM Single cell 5′ HT Reagent Kit v2.

##### Cross-tissue snRNA-seq (GTEx)

Sequencing reads for 25 samples belonging to 16 individuals from 8 tissue sites were downloaded from GTEx v9.

#### Alignment and CG quantification

Sequencing reads were aligned to the GRCh38 reference genome using STARsolo. The GRCh38 analysis set was indexed with the GENCODE v38 annotation to enable CG quantification. Parameters used for alignment included sample-specific settings (i.e., UMI length, cell barcode whitelist, etc.) that were selected depending on the reagent kit and chemistry. For all samples, we increased thresholds on the number of multimapping reads reported by the aligner (--outFilterMultimapNmax 500) and relaxed the score needed for suboptimal multimapping alignments to be reported (--outFilterMultimapScoreRange 5). Effectively, these settings increase the number of alignments reported for reads that map to multiple genomic locations, including alignments that may have been excluded due to sequencing or reference errors using the default settings. These additional multimappers provide Stellarscope with a more complete set of possible alignments over which to optimize the model. The alignments are output as a Binary Alignment Map (BAM) file with auxiliary tags indicating the cell barcode and UMI for each alignment record. UMI counts for CGs are output in MTX format.

#### Preprocessing

Quality control was performed on the data at the cell level. Scater^48^ functions were used to identify outliers in the percentage of mitochondrial reads, total number of features, and total number of molecules detected, distributions and remove cells using these adaptive thresholds. Cell type identity was predicted by reference mapping using Azimuth^31^ to the HuBMAP human PBMC ref. ^32^. Multiplets were detected using Scrublet^33^ and removed. The list of cell barcodes from cells that passed these filters was subsequently used for the Stellarscope analysis.

#### TE quantification with stellarscope

Alignments generated by STARsolo and the list of filtered cell barcodes were used as input to Stellarscope. First, Stellarscope filters the BAM file to include only alignments belonging to cells in the filtered barcode list and sorts the alignments by read name. The query name sorted BAM file is then intersected with the TE annotation to identify reads with alignments overlapping TE loci. PCR duplicates within these TE-overlapping reads are identified using the multimap-aware UMI deduplication algorithm. Transcript proportions, reassignment proportions, and alignment posterior probabilities are then estimated using the “celltype” pooling model. The final assignment for each read is determined using the “best_exclude” heuristic, where the single alignment with the highest posterior probability is used; if multiple alignments have equal highest posterior probabilities, the read is excluded.

##### TE count correction

Stellarscope outputs a TE count matrix with the number of UMIs assigned to each feature in the user-provided TE annotation. This is performed independently from other quantification procedures using different annotations. When the Stellarscope matrix is merged with other count matrices, i.e., gene counts from STARsolo, the matrices must be corrected to avoid having the same observation (read, UMI) contribute multiple counts to the final matrix. For example, if there is intersection between the exons of a TE and a canonical gene, and a read is aligned to the intersecting region, it may contribute one count to each of the matrices and thus two counts to the merged matrix. Correction consists of identifying reads or UMIs that are double-counted and removing the extra counts from one of the matrices.

Before merging the Stellarscope count matrix with the canonical gene matrix, we created a correction matrix containing, for each TE feature, the number of UMIs that were counted in the CG annotation. To determine whether a read was counted in the CG annotation, we used the “GX” tag from the BAM alignment record.

#### Visualization and identification of HERV markers

For each dataset, a merged matrix was created from the CG and TE UMI count matrices and subsequent analyses were performed using Seurat version 4.^32^ The combined UMI count matrix was normalized and transformed. Highly variable features (HVFs) were identified and used for dimensionality reduction and clustering. Markers were identified by differential expression testing among cell type identities and/or unsupervised clusters. Specific methods, statistical tests, and thresholds are described below.

#### Comparison of stellarscope with existing methods

We compared Stellarscope with two previously published software packages for TE quantification: scTE^24^ and soloTE.^25^ We reformatted the TE annotation in the required format for each program. Specifically, both programs expect a BED formatted file, and the “name” column is used to determine the TE classification of the genomic region. For scTE, the TE subfamily is used as the identifier, while soloTE uses a string encoding the repeat class, repeat family, subfamily, and locus. Both programs were run using the default settings recommended by the authors, and output files containing the estimated UMI counts. For soloTE, multiple outputs are generated including UMI counts at the subfamily level, locus level, and “legacy” level, which combines locus-specific and subfamily features.

In order to compare the estimates of scTE, soloTE, and Stellarscope, we calculated the upper and lower bounds on the UMI counts to determine whether the reported values were feasible. As this is empirical data, the true UMI counts (“ground truth”) are unknown; however, the expected upper and lower bounds on UMI counts could be determined by examining the alignment (BAM file) that is used as input for all three programs. To find the upper bound, the UMI count must be less than the number of distinct UMIs belonging to alignments that overlap with the TE regions. The lower bound is calculated with the same criteria but also requiring that reads are uniquely aligned. We calculated these bounds by parsing the BAM file and evaluating alignments according to whether they satisfy these criteria, then counting the number of distinct UMIs across the sample or within each cell or locus. UMI counts estimated by each of the three programs were compared to the expected bounds calculated from the alignments.

### Quantification and statistical analysis

TE UMI counts used in this study were reported by Stellarscope using the “celltype” pooling mode, with cell type identities determined by reference mapping (Azimuth^31^) to a human PBMC ref. ^32^. Default priors were used for π=0 and θ=200000. For model optimization, the EM algorithm was run for 500 iterations or until the change in parameter values was less than 1e-7. Final assignments for reads were determined using the “best_exclude” method which selects the single alignment with the greatest posterior probability or excludes the read if multiple alignments share the same greatest posterior probability. The reassigned read locations, cell barcodes, and UMIs were counted to create a TE UMI count matrix. A correction matrix was created by identifying reads that were included in both TE and CG UMI counts using the “GX” tag from BAM alignment records. The final TE UMI count matrix was created by subtracting the correction matrix from the initial TE UMI count matrix.

TE load for each cell was calculated by dividing the number of UMIs assigned to TE features by the total number of UMIs assigned to features; HERV and L1 load are calculated in the same manner (Figure 2D, S1, S2; 6A; 7A–7F). The distribution of TE load across cells was compared for different cell subtypes using a Kruskal Wallis rank-sum test; pairwise comparisons between subtypes were performed using a Wilcoxon Rank-Sum test, adjusted *p*-values <0.01 are shown (Figure 2D, S1, and S2).

Feature selection is based on residual variance (RV) calculated by scTransform^36^ (Figures 2G–2J; Figure 4A). Briefly, the residual variance for a feature is the variance of the Pearson residuals calculated by comparing observed values to those predicted by a regularized negative binomial regression model.^36^ Highly variable features (HVFs) were selected by choosing an RV threshold that included TE features; the same RV threshold was used for all feature types (CG, L1, HERV).

Dimensionality reduction is performed on transformed UMI counts for highly variable features (HVFs), or subsets, i.e., CG-HVF, HERV-HVF, L1-HVF (Figures 3, 4, 5, 6, and 7). First, the data is transformed using principal component analysis (PCA), storing the first 50 principal components (PCs). The most informative PCs are identified by selecting the first PC where the cumulative variance exceeds 90% and that contributes less than 5% of the variation (elbow method). The informative PCs are further reduced to 2 dimensions using uniform manifold approximation and projection (UMAP) implemented in umap-learn^50^^,^^51^ using the “RunUMAP” wrapper in Seurat.

Unsupervised clustering is performed using the informative PCs to generate nearest neighbor and shared nearest neighbor (SNN) graphs using the Seurat function “FindNeighbors”. SNN graphs are clustered using the Leiden algorithm^39^ implemented in leidenalg using the “FindClusters” wrapper in Seurat. We performed a sweep over the resolution parameter (0.2–1.6) and selected a resolution of 1.0 for visualization (Figures 3E–3G).

HERV markers were identified by differential expression testing using MAST^47^ implemented in the Seurat function “FindAllMarkers”. MAST is a generalized linear model framework that identifies enriched genes whilst correcting for covariates and gene-gene correlations. For a given cell type identity, we tested whether the UMI counts for that cell type were significantly different from all other cells in the population. Only features that were detected in >10% of cells from either population are tested, and tests with adjusted *p*-value <0.05 and average log_2_ fold change >0.25 were considered to be significant (Figures 4, 5, 6, and 7).

## Supplemental information

Document S1. Figures S1–S4

Document S2. Article plus supplemental information
