# Supplementary material for: A single-cell transposable element atlas of human cell identity
Source: Cell Rep Methods. 2025 Jun 20;5(7):101086. doi: 10.1016/j.crmeth.2025.101086 (PMC12296447; doi:10.1016/j.crmeth.2025.101086)
Supplement: Document S1. Figures S1–S4 [file mmc1.pdf]

Cell Reports Methods, Volume *5*

Supplemental information

A single-cell transposable element atlas of human cell identity

Helena Reyes-Gopar, Jez L. Marston, Bhavya Singh, Matthew Greenig, Jonah Lin, Mario A. Ostrowski, Kipchoge N. Randall Jr, Santiago Sandoval-Motta, Nicholas Dopkins, Elsa Lawrence, Morgan M. O'Mara, Tongyi Fei, Rodrigo R.R. Duarte, Timothy R. Powell, Enrique Hernández-Lemus, Luis P. Iñiguez, Douglas F. Nixon, and Matthew L. Bendall

Supplemental Figure Legends

Figure S1. HERV contribution to the transcriptome in PBMC cell subtypes, Related to Figure 2B-F.

The percentage of UMI counts that are assigned to HERV loci (left) and the number of HERV features detected (right). Individual cells are classified by mapping to the HuBMAP PBMC reference[45] at the celltype.l2 resolution using Azimuth[44]. Within each cell type – Dendritic cells, B cells, CD4+ T cells, CD8+ T cells, other T cells, NK cells, and other – the difference between the means of cell subtypes was tested using the Kruskal-Wallis test, and pairwise comparisons were tested using Wilcoxon rank-sum tests.

Figure S2. L1 contribution to the transcriptome in PBMC cell subtypes, Related to Figure 2B-F.

The percentage of UMI counts that are assigned to L1 loci (left) and the number of L1 features detected (right). Individual cells are classified by mapping to the HuBMAP PBMC reference[45] at the celltype.l2 resolution using Azimuth[44]. Within each cell type – Dendritic cells, B cells, CD4+ T cells, CD8+ T cells, other T cells, NK cells, and other – the difference between the means of cell subtypes was tested using the Kruskal-Wallis test, and pairwise comparisons were tested using Wilcoxon rank-sum tests.

Figure S3. Distribution of UMI counts per cell in 3' and 5' datasets, Related to Figure 6.

The number of UMI counts per cell is shown along the X axis and the percentage of cells is shown along the Y axis. 3' dataset shown in green, 5' dataset in orange.

Figure S4. Comparison of HERV markers in 3' and 5' datasets, Related to Figure 6D,E

Feature plots showing the relative expression level of HERV features in each cell for each significant HERV marker identified in the 3' datasets. Both datasets were projected into the

human PBMC reference UMAP space. Feature plots are titled with the feature name and the protocol used; within each plot, every cell is colored according to the scaled HERV expression detected in that cell. DE test results are shown in the lower left of each plot. For significant tests (plain typeface) the identity of the cell subset is shown with the average $\log_2$ fold change in parentheses. For non-significant tests (italics) the cell subset is shown and the average $\log_2$ fold change and adjusted p-value is shown in parentheses. Note that a test may meet thresholds (adjusted p-value < 0.05 and average $\log_2$ fold change > 0.25) but fail to meet detection threshold > 0.1 and thus be considered not significant. Text is colored according to the celltype color palettes.

Figure S1

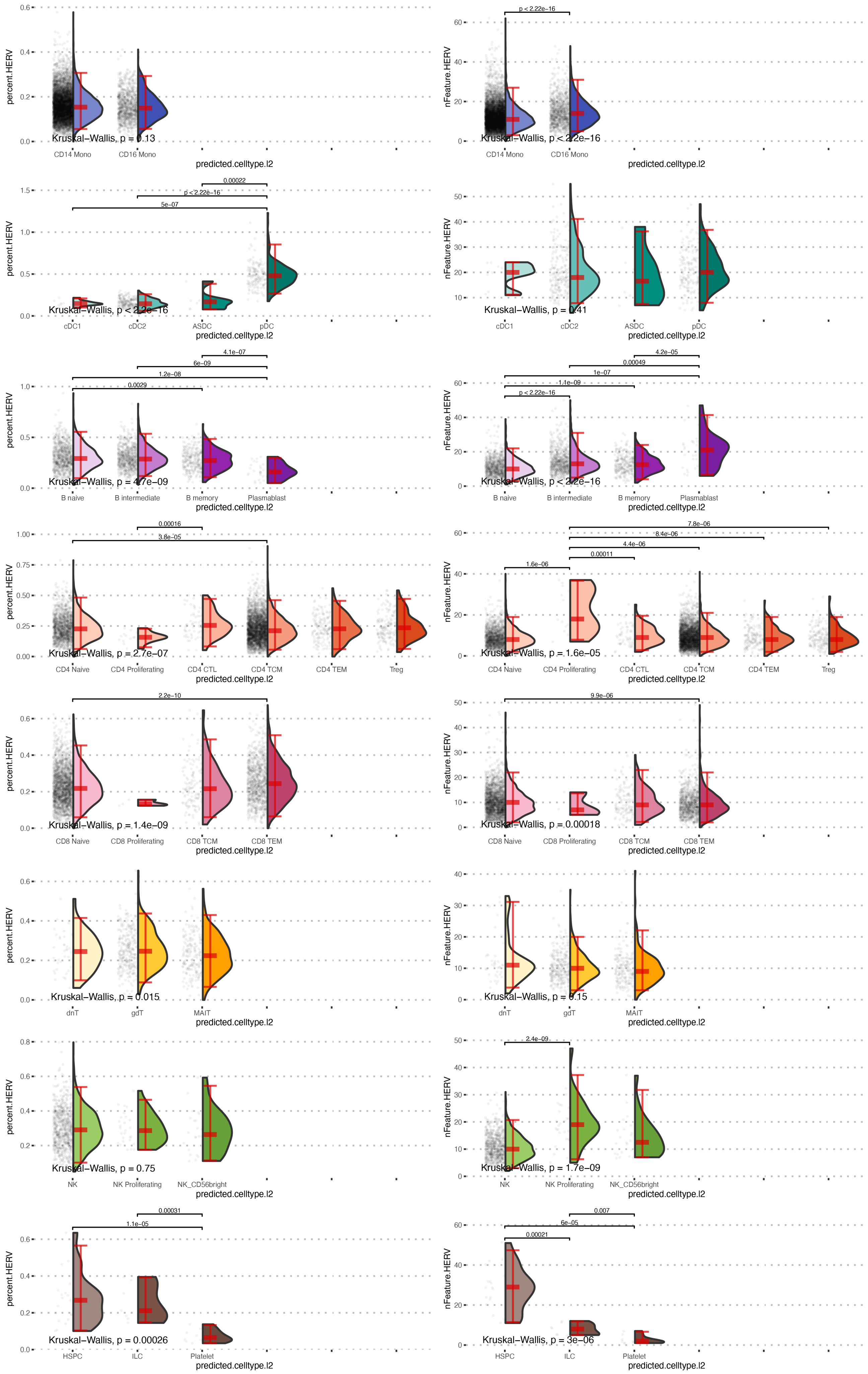
percent.HERV
nFeature.HERV
predicted.celltype.l2
CD14 Mono
CD16 Mono
Kruskal–Wallis, p = 0.13
Kruskal–Wallis, p < 2.2e−16
p < 2.22e−16
cDC1
cDC2
ASDC
pDC
Kruskal–Wallis, p < 2.2e−16
Kruskal–Wallis, p = 0.41
0.00022
p < 2.22e−16
5e−07
B naive
B intermediate
B memory
Plasmablast
Kruskal–Wallis, p = 4.7e−09
Kruskal–Wallis, p < 2.2e−16
4.1e−07
6e−09
1.2e−08
0.0029
4.2e−05
0.00049
1e−07
1.1e−09
p < 2.22e−16
CD4 Naive
CD4 Proliferating
CD4 CTL
CD4 TCM
CD4 TEM
Treg
Kruskal–Wallis, p = 2.7e−07
Kruskal–Wallis, p = 1.6e−05
0.00016
3.8e−05
7.8e−06
8.4e−06
4.4e−06
0.00011
1.6e−06
CD8 Naive
CD8 Proliferating
CD8 TCM
CD8 TEM
Kruskal–Wallis, p = 1.4e−09
Kruskal–Wallis, p = 0.00018
2.2e−10
9.9e−06
dnT
gdT
MAIT
Kruskal–Wallis, p = 0.015
Kruskal–Wallis, p = 0.15
NK
NK Proliferating
NK_CD56bright
Kruskal–Wallis, p = 0.75
Kruskal–Wallis, p = 1.7e−09
2.4e−09
HSPC
ILC
Platelet
Kruskal–Wallis, p = 0.00026
Kruskal–Wallis, p = 3e−06
0.00031
1.1e−05
0.007
6e−05
0.00021

Figure S2

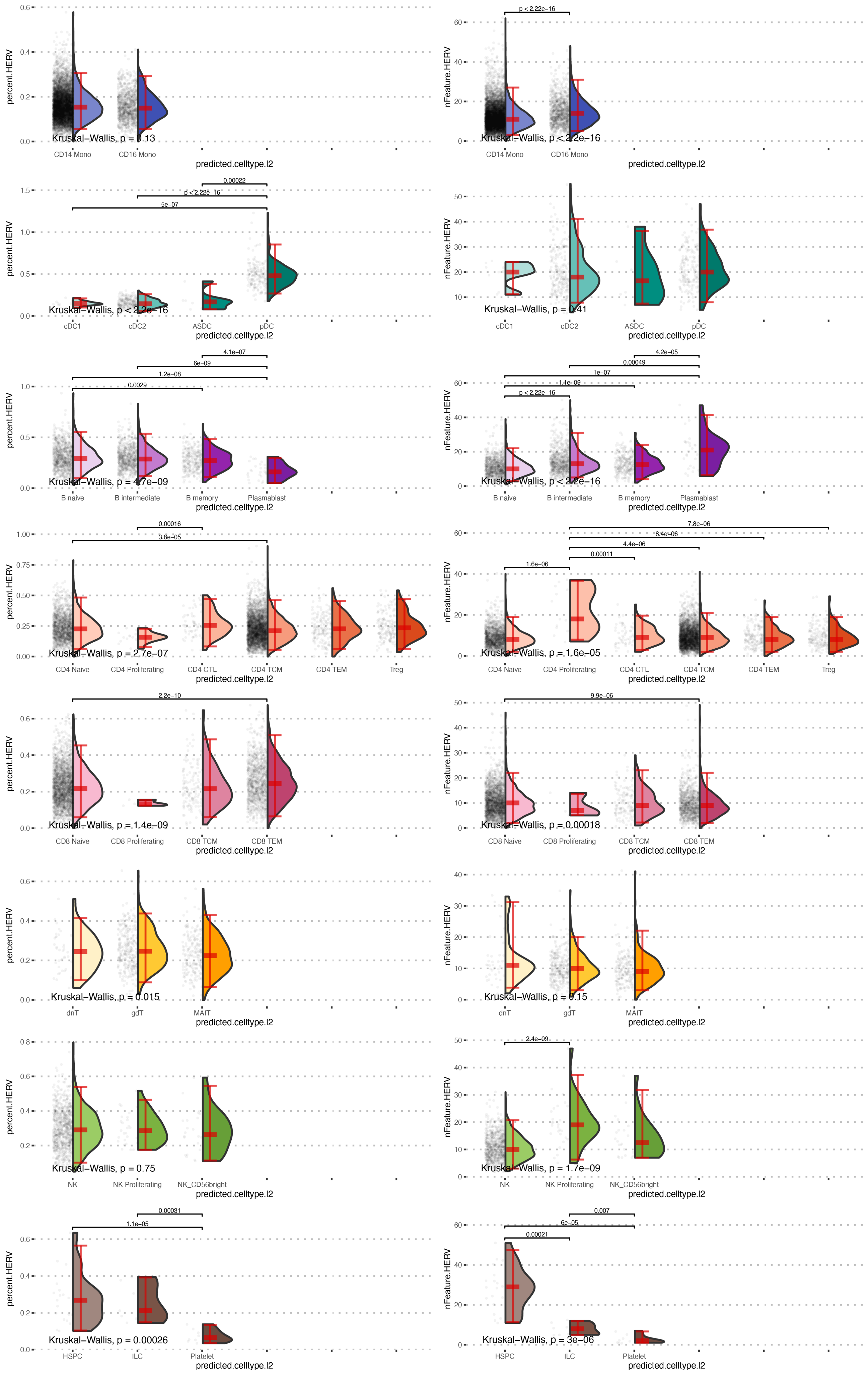

percent.HERV
nFeature.HERV
predicted.celltype.l2
Kruskal–Wallis, p = 0.13
Kruskal–Wallis, p < 2.2e−16
CD14 Mono
CD16 Mono
Kruskal–Wallis, p < 2.2e−16
Kruskal–Wallis, p = 0.41
cDC1
cDC2
ASDC
pDC
Kruskal–Wallis, p = 4.7e−09
Kruskal–Wallis, p < 2.2e−16
B naive
B intermediate
B memory
Plasmablast
Kruskal–Wallis, p = 2.7e−07
Kruskal–Wallis, p = 1.6e−05
CD4 Naive
CD4 Proliferating
CD4 CTL
CD4 TCM
CD4 TEM
Treg
Kruskal–Wallis, p = 1.4e−09
Kruskal–Wallis, p = 0.00018
CD8 Naive
CD8 Proliferating
CD8 TCM
CD8 TEM
Kruskal–Wallis, p = 0.015
Kruskal–Wallis, p = 0.15
dnT
gdT
MAIT
Kruskal–Wallis, p = 0.75
Kruskal–Wallis, p = 1.7e−09
NK
NK Proliferating
NK_CD56bright
Kruskal–Wallis, p = 0.00026
Kruskal–Wallis, p = 3e−06
HSPC
ILC
Platelet

Figure S3

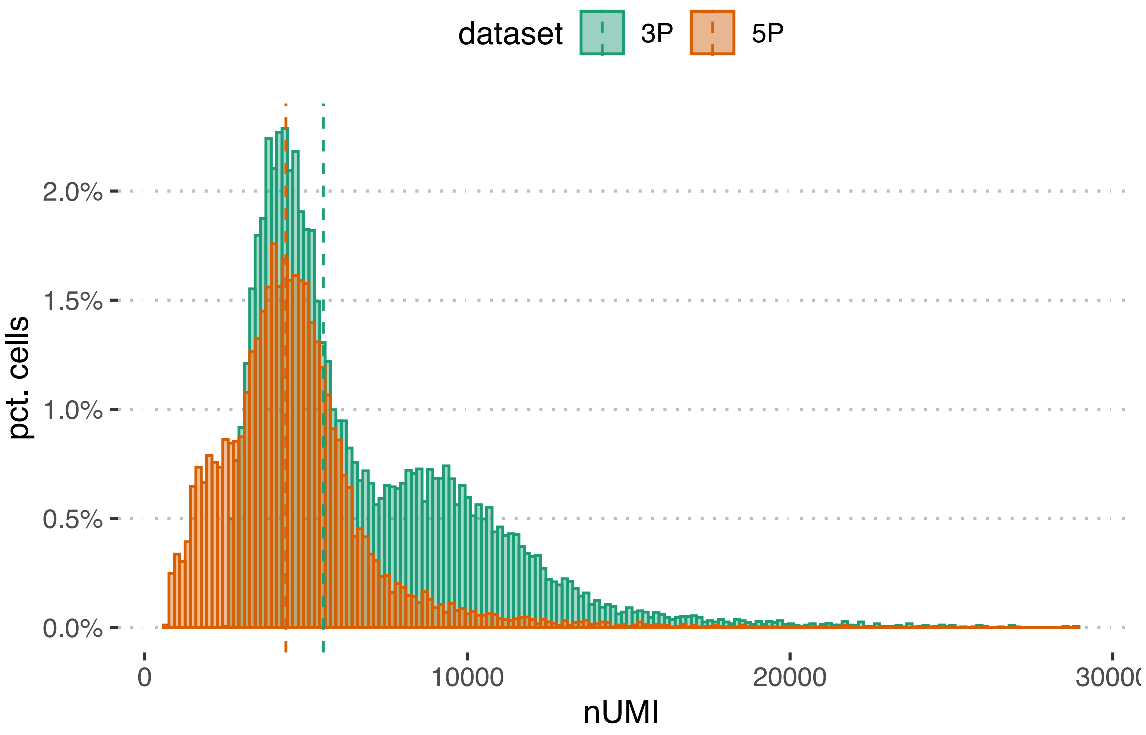

Figure S4

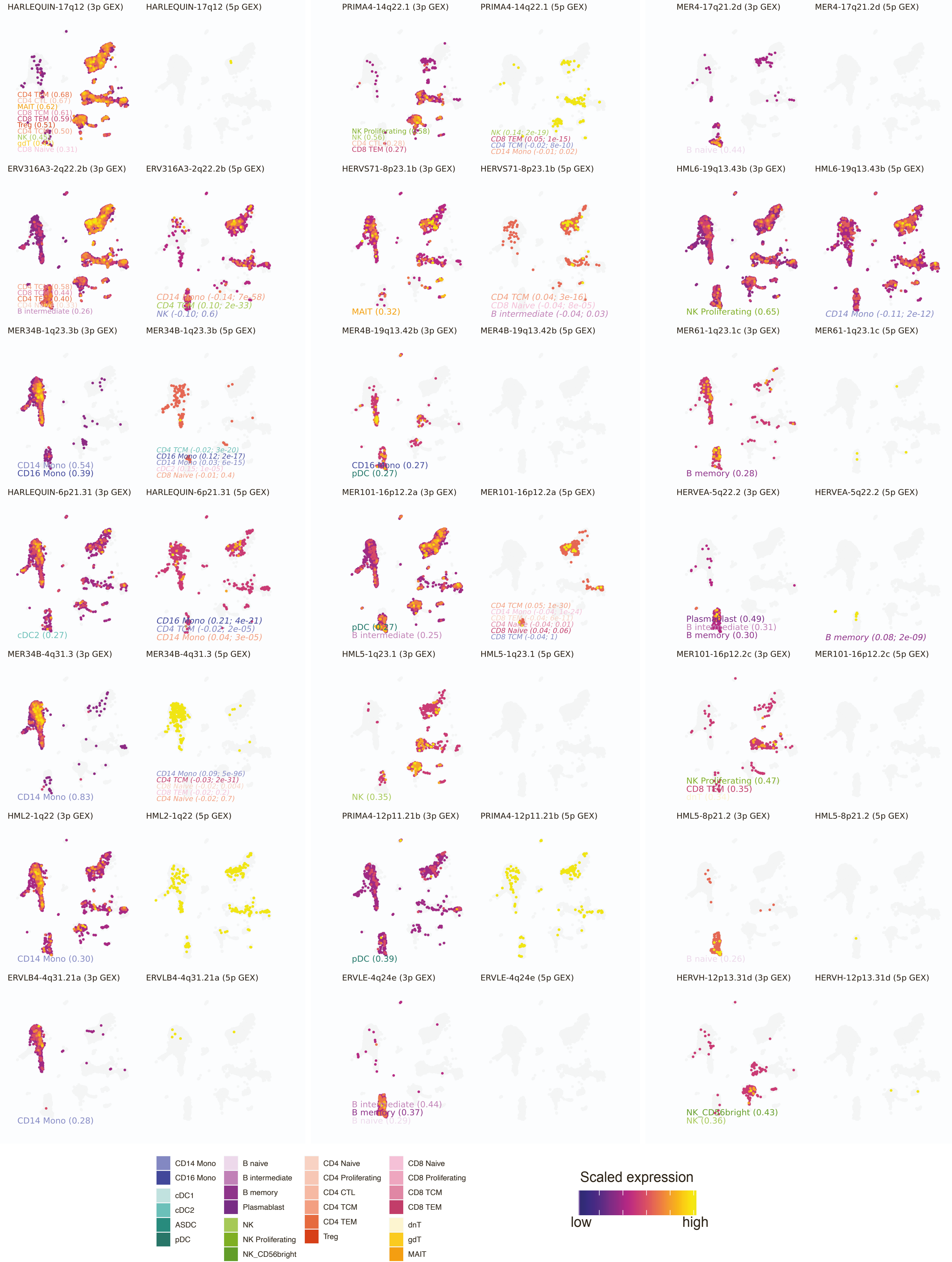
HARLEQUIN-17q12 (3p GEX)
HARLEQUIN-17q12 (5p GEX)
PRIMA4-14q22.1 (3p GEX)
PRIMA4-14q22.1 (5p GEX)
MER4-17q21.2d (3p GEX)
MER4-17q21.2d (5p GEX)
ERV316A3-2q22.2b (3p GEX)
ERV316A3-2q22.2b (5p GEX)
HERVS71-8p23.1b (3p GEX)
HERVS71-8p23.1b (5p GEX)
HML6-19q13.43b (3p GEX)
HML6-19q13.43b (5p GEX)
MER34B-1q23.3b (3p GEX)
MER34B-1q23.3b (5p GEX)
MER4B-19q13.42b (3p GEX)
MER4B-19q13.42b (5p GEX)
MER61-1q23.1c (3p GEX)
MER61-1q23.1c (5p GEX)
HARLEQUIN-6p21.31 (3p GEX)
HARLEQUIN-6p21.31 (5p GEX)
MER101-16p12.2a (3p GEX)
MER101-16p12.2a (5p GEX)
HERVEA-5q22.2 (3p GEX)
HERVEA-5q22.2 (5p GEX)
MER34B-4q31.3 (3p GEX)
MER34B-4q31.3 (5p GEX)
HML5-1q23.1 (3p GEX)
HML5-1q23.1 (5p GEX)
MER101-16p12.2c (3p GEX)
MER101-16p12.2c (5p GEX)
HML2-1q22 (3p GEX)
HML2-1q22 (5p GEX)
PRIMA4-12p11.21b (3p GEX)
PRIMA4-12p11.21b (5p GEX)
HML5-8p21.2 (3p GEX)
HML5-8p21.2 (5p GEX)
ERVLB4-4q31.21a (3p GEX)
ERVLB4-4q31.21a (5p GEX)
ERVLE-4q24e (3p GEX)
ERVLE-4q24e (5p GEX)
HERVH-12p13.31d (3p GEX)
HERVH-12p13.31d (5p GEX)
CD14 Mono
CD16 Mono
cDC1
cDC2
ASDC
pDC
B naive
B intermediate
B memory
Plasmablast
NK
NK Proliferating
NK_CD56bright
CD4 Naive
CD4 Proliferating
CD4 CTL
CD4 TCM
CD4 TEM
Treg
CD8 Naive
CD8 Proliferating
CD8 TCM
CD8 TEM
dnT
gdT
MAIT
Scaled expression
low
high
